# A novel anti *Candida albicans* drug screening system based on high-throughput microfluidic chips

**DOI:** 10.1038/s41598-019-44298-w

**Published:** 2019-05-30

**Authors:** Le Qiang, Jing Guo, Yingkuan Han, Jianfeng Jiang, Xiaowen Su, Hong Liu, Qingguo Qi, Lin Han

**Affiliations:** 1 Institute of Marine Science and Technology, Shandong University, 72 Binhai Road, Qingdao, 266237 China; 2 Endodontics, Taian Stomatology Hospital, 261-1 Lingshan Avenue, Taian, 271000 China; 3 School of Microelectronics, Shandong University, 27 South Shanda Road, Jinan, 250010 China; 4 State Key Laboratory of Crystal Materials, Shandong University, 27 South Shanda Road, Jinan, 250010 China; 5 Institute for Advanced Interdisciplinary Research, Jinan University, Jinan, 250022 China; 6 Cheeloo Health Science Center, Shandong University, 44 West Wenhua Road, Jinan, 250012 China

**Keywords:** Health care, Diseases

## Abstract

Due to the antibacterial resistance crisis, developing new antibacterials is of particular interest. In this study, we combined the antifungal drug amphotericin B with 50,520 different small molecule compounds obtained from the Chinese National Compound Library in an attempt to improve its efficacy against *Candida albicans* persister cells. To systematically study the antifungal effect of each compound, we utilized custom-designed high-throughput microfluidic chips. Our microfluidic chips contained microchannels ranging from 3 µm to 5 µm in width to allow *Candida albicans* cells to line up one-by-one to facilitate fluorescence-microscope viewing. After screening, we were left with 10 small molecule compounds that improved the antifungal effects of amphotericin B more than 30% against *Candida albicans* persister cells.

## Introduction

In recent decades, fungal infections have increased in severity. People are more at-risk for opportunistic fungal infections than ever, especially those who undergo organ transplants, prosthetic heart valve implants, or chemotherapy; those who use immunosuppressants or broad-spectrum antibacterials; those with HIV infections; premature infants; and the elderly^1–5^. At present, there is a very small variety of effective antifungal agents from which to choose. As a result, chronic, secondary fungal infections often prove fatal long before the primary disease^6,7^. The abuse of broad-spectrum antibacterials has led to the development of microbiological drug resistance, partially through the activity of persister cells. Persisters were first described by Joseph Bigger in 1944, in one of the first studies on the mechanism of penicillin’s action^8^. Bigger discovered that although penicillin lysed the cell walls in a growing population of *Staphylococcus spp*., a small number of cells persisted and survived. Lewis, K noted that these cells were not antibacterial-resistant mutants but dormant, non-dividing cells^9^. Michael D. LaFleur continued work on the topic and proposed the idea of *Candida albicans* persisters in 2006^10^. *Candida albicans* is the most common clinical fungal infection. It is both a component of normal human microflora and a dangerous opportunistic pathogen. Invasive candidiasis can grow deep in tissue and is generally chronic and persistent, especially in patients with human immunodeficiency. Systemic *Candida* infections have a surprisingly high mortality rate: 29–76%^11–13^. Yamashita Ichiro *et al*. first reported the flocculation gene *FLO8* in the Yeast Saccharomyces^14^. Fang Cao suggested that *FLO8* may function downstream of the cAMP/PKA pathway, and together with *EFG1*, regulates the expression of hypha-specific genes and genes that are important for the virulence of *Candida albicans*^15^. In 2009, our research group screened out kinase GIN4 and transcription factor *FLO8*. In this work, we constructed a gene fragment using green fluorescence proteins (GFPs) to target *FLO8* in normal *Candida albicans* and induce more persisters. This multiplication of *FLO8* persisters allowed for easy observation by fluorescence microscopy, which enabled the related new drugs screening based on GFPs expression.

However, the current selection of anti *Candida albicans* drug is extremely limited: fluconazole, amphotericin B, caspofungin, and terbinafine make up the bulk of them. Moreover, these antifungals are toxic and exhibit unpleasant side effects^16^. It is imperative that new antibacterials are developed to address these limitations, including ones that are effective against persisters. K. Lewis *et al*. has reported that combining fluconazole with small molecule compounds can improve activity against *Candida albicans*^17^. Here, we report an approach to improve the efficacy of anti *Candida albicans* drug by combining amphotericin B with 50,520 different small molecule compounds obtained from the Chinese National Compound Library, and developed a new microfluidic chip for conducting *Candida albicans* antifungal screening. The miniaturization brought forth by microfluidics generally allows shorter time to results, integrates sample preparation, and makes fluid handling portable^18^. Microfluidic chips were reported to conduct flow cytometric analysis of fluorescently stained cells from different organisms^19^, rapid detection and identification of bacteria^20^, fungal separation and PCR amplification^21^. Balaban *et al*. firstly used microfluidic-chip-like device to observe the persisters^22^.

**Figure 1:**
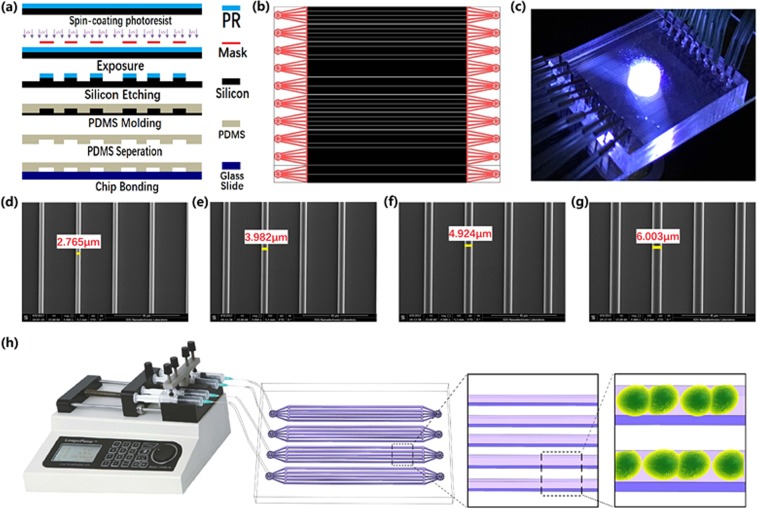
(**a**) The fabrication process of the drug-screening microfluidic chip; (**b**) The layout; (**c**) Photo of the microfluidic chip; (**d**) SEM microscope photo of the 3-µm microchannel; (**e**) The 4-µm microchannel; (**f**) The 5-µm microchannel; (**g**) The 6-µm microchannel; (**h**) System sketch. We thank Longer Precision Pump Co., Ltd. China for the authorization to use the pump photo.

Our microfluidic chips contain thousands of parallel microchannels through which many new drugs can be screened at the same time (Fig. 1). Microchannels are generally only a few micrometers wide, which allows a working volume of mere nanoliters by reducing reagent consumption to 1/100000 of traditional 384w-MTP^23–27^. Using our microfluidic chips, we performed the screening of 50,520 new drugs, and finally found 10 small molecule compounds that enhance amphotericin B’s anti-*Candida-albicans* effects against persisters by more than 30%^28,29^.

## Results

### New anti candida albicans drugs development

Previously, our team located the *FLO8* gene, which is related to the activity of *Candida albicans* persisters^16^. To target *FLO8*, we constructed a gene fragment with GFP. We then transferred this gene fragment to *Candida albicans* 3153A to obtain more *Candida albicans* persisters, and to make them readily observable using fluorescent microscopy.

To find new drug candidates, we selected 50,520 small molecule compounds from the Chinese National Compound Library and systematically combined each of them with amphotericin B. The resulting concentration of each new amphotericin B combination was 3.5 μg/mL. Suspended cells were injected into 10 microfluidic chips at the same time, and it took 48 hours for the cells to grow into biofilms. 100 compounds could be screened in one chip, and 20 chips were processed each time. The images of cells were taken under fluorescence microscope at 24 hours after drugs were loaded. The drug efficacy was evaluated by counting the alive persister cells in the microchannels.

### Microfluidic chips design and fabrication

The microchannels of microfluidic chip was designed based on the size of Candida albicans. The width and depth of microchannels are in the range of 3 µm–6 µm in order to let Candida albicans pass through and line up one by one. One chip has 20 units, each unit include tweenty 3 µm-microchannels, tweenty 4 µm-microchannels, tweenty 5 µm-microchannels, and tweenty 6 µm-microchannels. Each unit has an inlet and an outlet. The layout is shown in Fig. 1(a), and the fabrication process schematic is illustrated in Fig. 1(b). The silicon wafer with microchannels then was used hard mode for PDMS chips. The SEM views of microchannels in different sizes are shown in Fig. 1(d–g). The PDMS chip and a pre-cleaned glass slide were treated with oxygen plasma, it was immediately brought into contact against the slide to form closed channels. The microscope picture of the whole chip is as shown in Fig. 1(c), and the microfluidic platform was setup as shown in Fig. 1(h).

**Figure 2:**
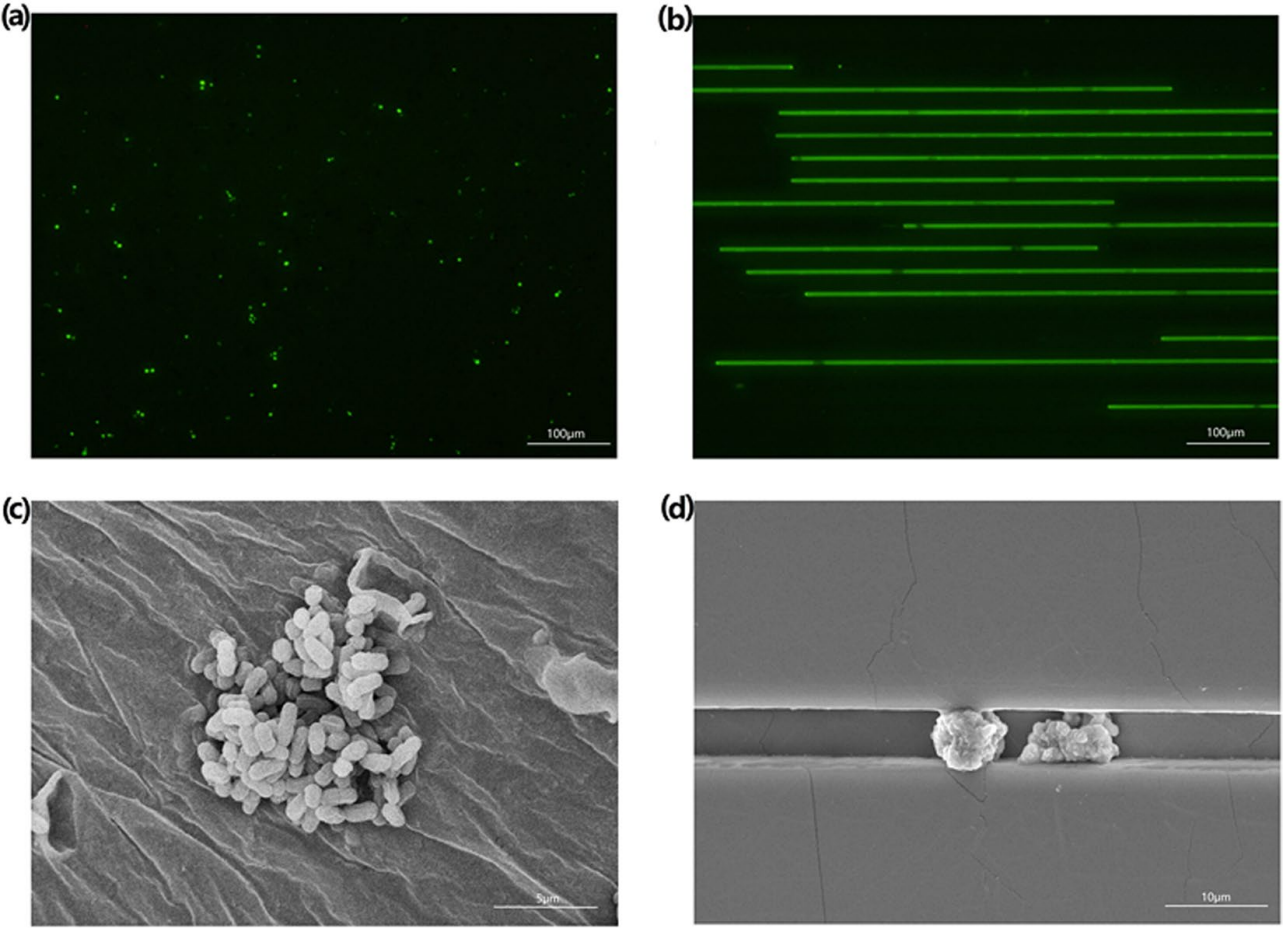
The comparison of *Candida albicans* in traditonal culture and microfluidic chips. (**a**) Fluorescence microscope view of normal *Candida albicans*; (**b**) Fluorescence microscope view of *Candida albicans* in microchannels; (**c**) SEM view of normal *Candida albicans*; (**d**) SEM view of *Candida albicans* in microchannels.

Figure 2(a,c) show the fluorescent and SEM microscope views of cells cultured in Petri dish, and Fig. 2(b,d) are those of cells in microchannels. In our study we discovered that 4-μm and 5-μm microchannels were most suitable for the growth of *Candida albicans*, while 3-μm microchannels were too narrow to allow for *Candida albicans* cell passage. Due to the obstructions created by the 3-μm microchannels, it was difficult for the pump to load the culture solution. Conversely, the 6-μm microchannels were too wide for the majority of *Candida albicans* cells and created cell overlap, making persister visualization difficult. Cells grow up in piles in the culture Petri dish, while they line up one by one in order in the microchannels. It showed similar growth curves in microchannels compared to traditional 384-well plate.

### New drug candidates found using high-throughput microfluidic chip screening

**Figure 3:**
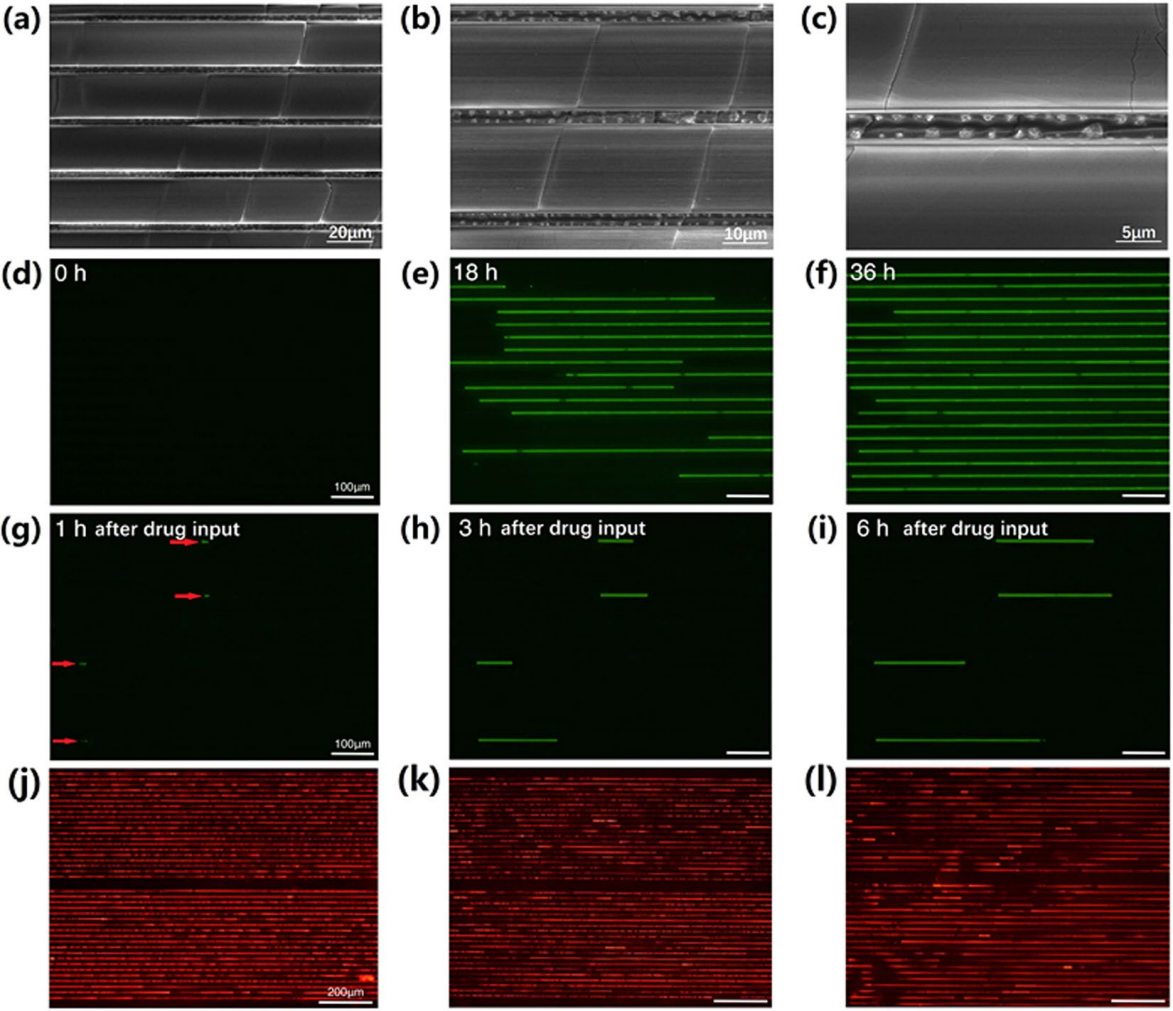
*Candida albicans* cultured in a microfluidic chip as seen under SEM at different magnifications (**a**–**c**) and fluorescent microscopy at (**d**) t = 0, (**e**) t = 18 h, (**f**) t = 36 h. One hour after new drug import into microchannels, *Candida albicans* persisters in a microfluidic chip under fluorescent microscopy at (**g**) t = 1 h, (**h**) t = 3 h, and (**i**) t = 6 h (The four red arrows identify the persisters.) *Candida albicans* LIVE/DEAD® staining after drug exposure in a microfluidic chip at (**j**) t = 1 h, (**k**) t = 3 h, and (**l**) t = 6 h.

The *Candida albicans* suspension was loaded into a 1- ml injector, and an injection pump was used to inject the solution into microfluidic chips at a rate of 5 μm/min. 100 small molecules and amphotericin B mixtures were screened in each chip, and 20 microfluidic chips were conducted at the same time. Adhesive *Candida albicans* settled down and spreaded on the PDMS channel surface in 2 hours, and Fresh RPMI-1640 was provided every 6 hours up to 36 hours, at which point the microchannels had been completely filled by growing *Candida albicans* in line (Fig. 3a–c). Then 2 μL of a drug solution was loaded into each microfluidic chip inlet, and the microchannels were filled via vacuum suction at the outlet. Due to the adhesive characteristics of *Candida albicans* cells, the cells remained in the microchannels throughout the culture and drug-input process. After the cells were exposed to the drugs for 1 hour, fresh RPMI-1640 culture medium was loaded into the injection pump to flush out the drug. The microfluidic chip was then observed under fluorescent microscopy to check for persisters (Fig. 3d–f).

GFP constructed in *FLO8* is only active when cells are alive. However, in order to confirm *Candida albicans* cell death, LIVE-DEAD^®^ staining was used to verify a compound’s fungicidal effects. After incubation, the microchannels were observed under fluorescent microscopy (Fig. 3g–l). Green fluorescent indicated live cells, and red fluorescent indicated dead cells. This method made it easy to observe whether or not the drug compounds exhibited a fungicidal effect on *Candida albicans*.

To construct an evaluation equation, we considered the number of *Candida albicans* persisters per unit of microchannel length.

$$n=\sum \frac{(pl)i}{al}\times \frac{1}{L}$$

Here, *n* refers to the number of live *Candida albicans* persisters per unit length after exposed to drugs, *pl* is the total length of *Candida albicans* persisters under fluorescent microscope, and *al* represents the average length of a *Candida albicans* cell, which is usually 4.5 μm. *L* is the total length of microchannels, and each microchannel is 2 cm long.

**Figure 4:**
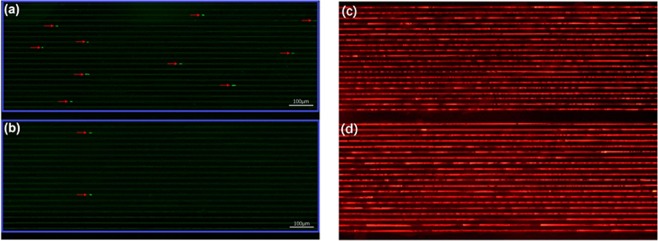
Surviving *Candida albicans* persisters after treatment with (**a**) Normal amphotericin B and (**b**) Amphotericin B in combination with small molecule compounds. The red arrows point to the persisters. (**c**,**d**) Are the LIVE-DEAD® fluorescent images corresponding to (**a**,**b**).

Fungicidal effects were evaluated based on the number of surviving *Candida albicans* persisters remaining in the microchannels. 20 replicates were done for each drug, and the average value of 20 tests was used to describe the final efficacy. We observed significant efficacy differences between amphotericin B by itself versus the amphotericin B and small molecule combinations (Fig. 4).

**Figure 5:**
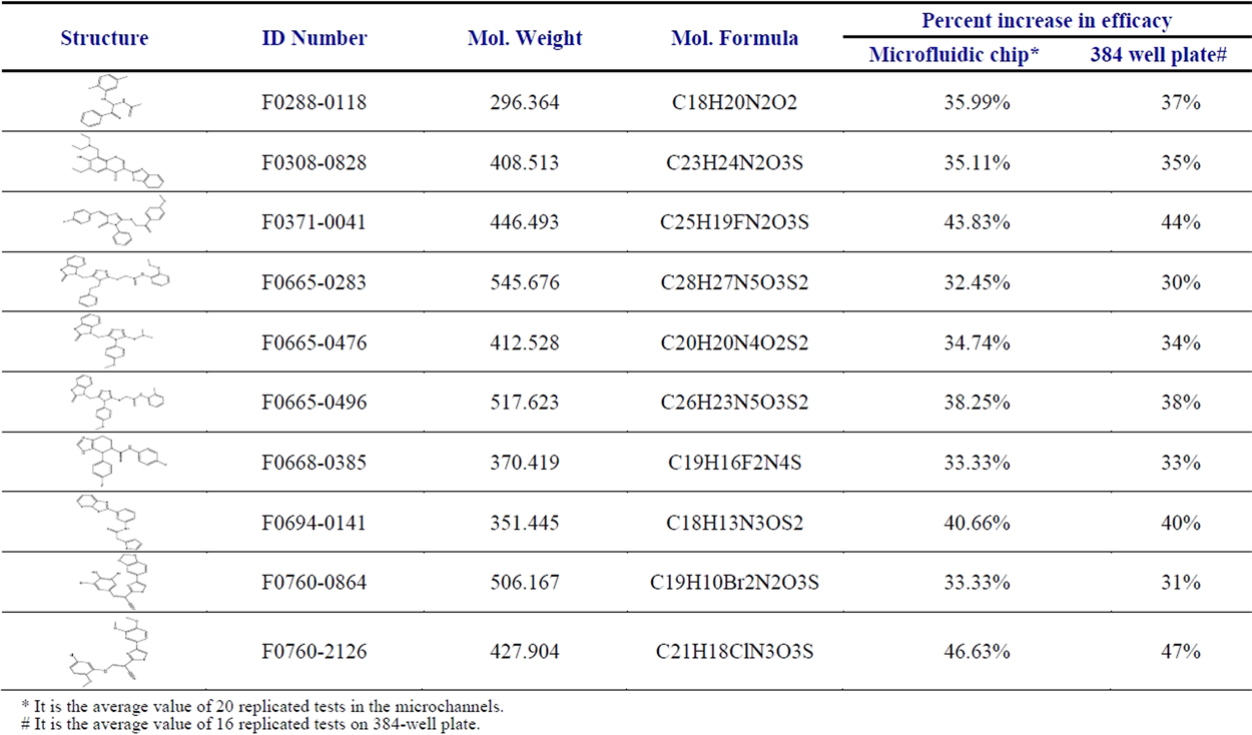
10 small molecule compounds improve the fungicidal effect of amphotericin B by more than 30% against *Candida albicans* persister cells.

Using high-throughput screening, we discovered 10 small molecule compounds (out of 50,520) that increase amphotericin B’s fungicidal effect on *Candida albicans* persisters by over 30% (Fig. 5). Our tentative hypothesis is that the 10 amphotericin B combinations displayed increased efficacy because of the presence of azolates and azolate derivatives. Compounds 2,4,5,6, and 9 contain benzothiazole structures (Fig. 5) while compounds 4,5, and 6 contain 3 azole structures. Compound 3 and 7 are imidazoles. Compound 9 and10 are thiazoles. Several of the most potent clotrimazole potentiators contain a 1,3-benzothiazole scaffold (compounds 2,4,5,6 and 9)^17,30^. The antifungal properties of benzothiazole compounds have been reported^31^. One example is 6-amino-2-n-pentylthiobenzothiazole, which has been found to inhibit Candida filamentation^32^. Unfortunately, some benzothiazoles exhibit cytotoxicity, such as 1,3-benzothiazole. However, such a liability may be irrelevant for topical applications. Alternatively, it may be possible to reduce the scaffold’s cytotoxicity by synthesizing and testing chemical analogues^17^. Other azole potentiators have been found to function via diverse mechanisms, including inhibiting calcineurin^33^, HSP90^34,35^, and drug efflux pumps^36,37^.

It is also possible that the amphotericin B combinations contain conjugated systems containing nitrogen and sulfur atoms which enhance the fungicidal effect. Two examples of π-π conjugated systems are CH_2_=CH-CH=O and CH_2_=CH-C≡N. The presence of such conjugations may alter amphotericin B’s three-dimensional structure. The altered structure may inhibit *Candida albicans*’ drug efflux pump, thereby keeping amphotericin B in the cell.

## Discussion

The development of new drugs is a lengthy process, and high-throughput microfluidic platform screening is an innovative method that can be utilized to advance drug research. It’s a highly-sensitive method that provides rapid, accurate results. In addition, its setup is compact. As shown in Table 1, the highly integrated microchannels enabled microfluidic chips to process over 1000 kinds of drugs simultaneously, and 50520 drugs could be screened in one week. Traditional HTS tests the efficacy percentage by Alamar Blue, which based on oxidation-reduction reaction. It is tedious to calculate the absorbance at 570 mm and 600 mm wavelengths with complicated steps^38^. The microfluidic chips ensure the passage of *Candida albicans* that pass through and line up one-by-one in the microchannels, which makes the drug evaluation more convenient and accurate because of the straightforward calculation and detection of small probability of residual bacteria.

**Table 1:** Comparison of our custom-designed microfluidic chips and traditional HTS methods.

	Microfluidic chips	Traditional HTS Methods (384w-MTP as example)^39^
Principle	Direct living cells counting	AlamarBlue Redox method, or 3- (4,5-dimethylthiazol-2-yl)-2,5-diphenyl tetrazolium bromide (MTT) method^40,41^, Indirect method
Quantity	100 kinds of drug	20 kinds of drug
Operation time for 50520 drugs screening	1 week	125 days
Observation	Visually, intuitively	Indirectly
Reagent	Totally <1 μL	>100 μL/well

## Conclusion

Through our high-throughput microfluidic chips, we have erected a novel platform for *Candida albicans* antifungal drug screening. We were able to screen 50,520 potential drugs easily and accurately. Ten of these compounds improved the efficacy of the anti-*Candida albicans* drug amphotericin B, increasing it by more than 30%. This discovery may contribute to the clinical treatment of refractory, recurrent *Candida albicans* infections and may also create a new path for drug development.

## Materials and Methods

### Cell culture and Regents

*Candida albicans* 3153A is wildtype strained. The *FLO8* plasmid with GFP was purchased in BioSune Biology Company, Shanghai, China. Stock cultures of *Candida albicans* strains were routinely cultured in YPD (1% yeast extract, 2% peptone, 2% glucose) solid medium containing 1.5% agar at 37 °C for 24 to 48 h. *Candida albicans* Yeast inocula cells were prepared by transferring a single colony into YPD medium with overnight incubation at 37 °C in an incubator shaker at about 100 rpm. Cells were harvested by centrifugation at 6,000 g for 3 min and washed twice in sterile phosphatebuffered saline (PBS [pH 7.2 to 7.4]). Then the cells were resuspended in RPMI 1640 medium (Gibco) with L-glutamine, buffered to pH 7.0 with 0.165 M morpholinepropanesulfonic acid (MOPS [Sigma-Aldrich]) and adjusted to the desired density of 2 × 10^3^~1 × 10^4^ ^42^. CLSI M27-A3 microdilution methodology was used to test *in vitro* susceptibility to amphotericin B(Amresco)^43^. LIVE-DEAD^®^ Funga Light yeast viability kit was purchased from Invitrogen. The link for 50,520 different small molecule compounds is: http://www.cncl.org.cn/.

### New anti candida albicans drugs preparation

The initial concentration of each small molecule compound solution (dissolved in DMSO) was 1000 µg/mL and total of 1 μL. In order to ensure the maximum concentration of dissolved <1% compounds and the DMSO final effect on *Candida albicans*, we added in the original compounds on the basis of 2 μL DMSO and 7 μL RPMI-1640 dilution of small molecules compound. We then mixed 10 μL of each small-molecular-compound with 100 μL of 2-μg/mL amphotericin B, following the NCCLS M27-A2 microdilution method^44^. The resulting concentration of each new amphotericin B combination was 3.5 μg/mL. We use injection pump (Longer Precision Pump Co., Ltd. China) to inject new drugs.

### PDMS microfluidic chips fabrication

The microfluidic chip was fabricated by multilayer soft lithography technique using polydimethylsiloxane (PDMS; Sylgard 184A and B). One photomask was first generated with microscal patterns designed by computer-aided design software L-Edit and transferred on Cr mask substrate. The positive photoresist AZ5214 was spun on a 6-inch silicon wafer, and got exposed under UV light with Cr mask on top. After the photoresist was development, the silicon wafer was heated at 120 °C for 5 minutes to remove the moisture, and then Oxford 180 dry etcher was used to etch the silicon channels. After silicon etching, the photoresist was removed by acetone and Isopropanol. The silicon wafer with microchannels then was used hard mode.

To make a PDMS chip, a ratio of Sylgard A:B = 10:1 by weight was mixed thoroughly and poured onto the silicon mold in a Petri dish at a thickness of approximately 5 mm. After all of the bubbles in the PDMS were vacuumed out, the Petri dish containing the PDMS was cured at 80 °C for 60 minutes. Then the PDMS layer was then peeled off of the mold and perforated to produce inlets and outlets for cell solution and medicine loading. Both the PDMS chip and clean glass slide were treated with oxygen plasma at 50 W for 5 minutes, and then they were lined up and bonded pemamently.

### SEM sample preparation

We referred to the publication by W. Krzysciak to prepare the SEM sample preparation^45^ PDMS microfluidic chip was separated from the glass substrate, and *Candida albicans* cells were kept in the microchannels because they were adhesive. The PDMS chip was washed three times with 0.1 M PBS, and treated with 1% osmium tetroxide for 1 h at room temperature. Then the PDMS was fixed with 2% glutaraldehyde for 2 h at room temperature. The PDMS was dehydrated using graded ethanol (10%, 30%, 50%, 70% and 100%) and putted in vacuum freeze-drying machine for 8 hours. Finally, the PDMS was mounted on microscope stubs and sputtered with a thin layer of gold, and then examined using a Leica S440 scanning electron microscope.

### Comments

The photo of injection pump in Fig. 1h has been provided by Longer Precision Pump Co., Ltd. China, where we bought the injection pump. We got the company’s authorization to use this photo.
